# A Comparative Analysis of Pricing and Reimbursement of Cystic Fibrosis Transmembrane Conductance Regulator Modulators in Europe

**DOI:** 10.3389/fphar.2021.746710

**Published:** 2021-11-08

**Authors:** Khadidja Abdallah, Kris De Boeck, Marc Dooms, Steven Simoens

**Affiliations:** ^1^ Department of Pharmaceutical and Pharmacological Sciences, KU Leuven, Leuven, Belgium; ^2^ Department of Pediatrics, KU Leuven, Leuven, Belgium

**Keywords:** cystic fibrosis, gene modulators, pricing, reimburesement, comparative analaysis

## Abstract

**Objectives:** Cystic fibrosis transmembrane conductance regulator (CFTR) modulators, Kalydeco® (ivacaftor), Orkambi® (lumacaftor/ivacaftor) and Symkevi® (tezacaftor/ivacaftor), have substantially improved patients’ lives yet significantly burden healthcare budgets. This analysis aims to compare pricing and reimbursement of aforementioned cystic fibrosis medicines, across European countries.

**Methods:** Clinical trial registries, national databases, health technology assessment reports and grey literature of Austria, Belgium, Denmark, France, Germany, Ireland, Poland, Spain, Sweden, Switzerland, Netherlands, the United Kingdom were consulted. Publicly available prices, reimbursement statuses, economic evaluations, budget impact analyses and managed entry agreements of CFTR modulators were examined. Results: In Belgium, lowest list prices were observed for Kalydeco® (ivacaftor) and Symkevi® (tezacaftor/ivacaftor) at €417 per defined daily dose (DDD) and €372 per average daily dose (ADD), respectively. Whereas, Switzerland had the lowest price for Orkambi® (lumacaftor/ivacaftor) listed at €309 per DDD. Spain had the highest prices for Kalydeco® (ivacaftor) and Symkevi® (tezacaftor/ivacaftor) at €850 per DDD and €761 per ADD, whereas Orkambi® (lumacaftor/ivacaftor) was most expensive in Poland at €983 per DDD. However, list prices were subject to confidential discounts and likely varied from actual costs. In all countries, these treatments were deemed not to be cost-effective. The annual budget impact of the CFTR modulators varied between countries and depended on factors such as local product prices, size of target population, scope of costs and discounting. However, all modulators were fully reimbursed in ten of the evaluated countries except for Sweden and Poland that, respectively, granted reimbursement to one and none of the therapies. Managed entry agreements were confidential but commonly adopted to address financial uncertainties.

**Conclusion:** Discrepancies concerning prices, reimbursement and access were detected for Kalydeco® (ivacaftor), Orkambi® (lumacaftor/ivacaftor) and Symkevi® (tezacaftor/ivacaftor) across European countries.

## Introduction

Cystic fibrosis (CF) is a rare condition affecting more than 48,000 individuals in Europe. With an occurrence of 1 in 2000–3,000, it is also the continent with the highest incidence of CF (European Cystic Fibrosis Society, 2020) (Farrell, 2008; Bell et al., 2020). Over time, technological advancements such as preconception carrier screening have led to a decline in incidence rates in some countries or regions (Lopes-Pacheco, 2016; Bell et al., 2020). However, newborn screening, improved care and clinical awareness have contributed to decreased pediatric mortality, a stable and a continuously growing CF adult population, now exceeding the pediatric population (Burgel et al., 2015; Lopes-Pacheco, 2016; Balfour-Lynn and King, 2020; Bell et al., 2020).

Inheritance of the disease is autosomal recessive and caused by errors in the cystic fibrosis transmembrane conductance regulator (*CFTR*) gene (Rafeeq and Murad, 2017; Bell et al., 2020). Over 2000 *CFTR* mutations have been identified and are grouped into six classes based on the protein defect (Rafeeq and Murad, 2017). Class I mutations result in no functional CFTR and include nonsense mutations, splice mutations or deletions (De Boeck et al., 2014; Rafeeq and Murad, 2017). In Class II, characterized by the most common heterozygous or homozygous F508del mutation affecting 85% of people with CF (PWCF) in Europe, the CFTR protein is misfolded and unable to reach the cell surface (De Boeck et al., 2014; Rafeeq and Murad, 2017). Gating mutations, typically describing G551D, S549R or V520F alterations that prevent opening of the CFTR channel, are categorized in Class III (De Boeck et al., 2014; Rafeeq and Murad, 2017). Class IV describes impairment of CFTR regulation by faulty channel conformation e.g. D1152H or R117H mutations (De Boeck et al., 2014; Rafeeq and Murad, 2017). Splicing mutations of Class V, such as 3,849+10 kb C → T, result in insufficient CFTR channels and Class VI mutations cause increased degradation of the unstable protein (De Boeck et al., 2014; Rafeeq and Murad, 2017).

A dysfunctional CFTR protein generates a chloride and bicarbonate ionic imbalance while increasing influx of sodium and water (Morrison et al., 2019). This disrupts the natural pH and alters the apical liquid layer of epithelial cells and digestive fluids into accumulating thick mucus or ‘mucoviscidosis’. This phenotypically manifests into persistent obstruction and inflammation of organs such as the lungs and gastrointestinal tract (NICE, 2017; Rafeeq and Murad, 2017; Morrison et al., 2019; Bell et al., 2020). Further complications can lead to deterioration of vital organs and death.

However, innovative therapies have increased life expectancy of PWCF to above 40 years (Lopes-Pacheco, 2016; Lopes-Pacheco, 2019). CFTR modulators have revolutionized the treatment of CF from symptomatic therapy, consisting of antibiotics, bronchodilators and mucolytic medicines, to mechanism-targeting therapies (Lopes-Pacheco, 2016; Cystic Fibrosis Foundation, 2021b). Currently, four modulators, developed by Vertex Pharmaceuticals, Inc., are authorized in the European Union, namely: Kalydeco^®^ (ivacaftor), Orkambi^®^ (lumacaftor/ivacaftor), Symkevi^®^/Symdeko^®^ (tezacaftor/ivacaftor) and Kaftrio^®^/Trikafta^®^ (ivacaftor/tezacaftor/elexacaftor) (European Medicines Agency, 2021b; European Medicines Agency, 2021c; European Medicines Agency, 2021d; European Medicines Agency, 2021a). Kalydeco^®^ (ivacaftor), the first CFTR potentiator introduced in 2012, is used in infants aged 4 months or older (European Medicines Agency, 2021b). Its active substance, ivacaftor extends the opening of the CFTR channel gate and increases activity of defective protein (Rafeeq and Murad, 2017; Lopes-Pacheco, 2019). Subsequently, Orkambi^®^ (lumacaftor/ivacaftor) was launched as a combination therapy for patients 2 years and older, with a homozygous F508del mutation, and contains both ivacaftor and lumacaftor (Lopes-Pacheco, 2016; Lopes-Pacheco, 2019; European Medicines Agency, 2021c). The latter corrects the misfolding of the CFTR protein and, in combination with potentiator Kalydeco^®^ (ivacaftor), facilitates chloride secretion. Symkevi^®^ (tezacaftor/ivacaftor) is indicated in patients aged 6 years and older with the F508del mutation, homozygous or heterozygous with a residual function mutation (Lopes-Pacheco, 2019; European Medicines Agency, 2021d). This therapy combines ivacaftor and tezacaftor and has clinically improved tolerability and pharmacokinetics than its predecessor Orkambi^®^ (lumacaftor/ivacaftor). Most recently, Kaftrio^®^ (ivacaftor/tezacaftor/elexacaftor) was approved for patients, 12 years or older homozygous or heterozygous with a minimal function mutation for the F508del mutation (Lopes-Pacheco, 2019; European Medicines Agency, 2021a). It is a triple combination therapy containing ivacaftor, tezacaftor and a third corrector, elexacaftor proven to be more efficacious than Symkevi^®^ (tezacaftor/ivacaftor). All these therapies, except for Orkambi^®^ (lumacaftor/ivacaftor), of which orphan designation was withdrawn at market authorization upon request of the company, are designated as orphan medicinal products (OMP). Symkevi^®^ (tezacaftor/ivacaftor) and Kaftrio^®^ (ivacaftor/tezacaftor/elexacaftor) are used in combination with Kalydeco^®^ (ivacaftor) in therapy (European Medicines Agency, 2021b; European Medicines Agency, 2021d; European Medicines Agency, 2021a).

Moreover, for each indication of Kalydeco^®^ (ivacaftor), Orkambi^®^ (lumacaftor/ivacaftor) and Symkevi^®^ (tezacaftor/ivacaftor), phase 3 clinical trials reported improved pulmonary functions, expressed in lung clearance index (LCI 2.5) and percentage predicted forced expiratory volume in one second (ppFEV_1_), compared to placebos (Table 1) (EU Clinical Trials Register, 2015b; EU Clinical Trials Register, 2015c; EU Clinical Trials Register, 2015a; EU Clinical Trials Register, 2017c; EU Clinical Trials Register, 2017b; EU Clinical Trials Register, 2017a; EU Clinical Trials Register, 2018). However, statistically significant difference was only achieved in 3,849 + 10 KB C→T or D1152H *CFTR* mutations, G551D and Non-G551D *CFTR* gating mutations for Kalydeco^®^ (ivacaftor), in homozygous F508del *CFTR* mutations for Orkambi^®^ (lumacaftor/ivacaftor) and Symkevi^®^ (tezacaftor/ivacaftor) (EU Clinical Trials Register, 2015c; EU Clinical Trials Register, 2015a; EU Clinical Trials Register, 2017c; EU Clinical Trials Register, 2017b).

**TABLE 1:** Description of design and efficacy results of the pivotal trials in each indication of Kalydeco^®^, Orkambi^®^ and Symkevi^®^.

Intervention	Indication	Design	Efficacy results:		
			primary endpoints (change from baseline)		
			Intervention	Placebo	Placebo vs intervention
					Parameter estimates and/or
					Statistical analysis
Kalydeco^®^ (ivacaftor)	3849 + 10KB C→T or D1152H-CFTR mutation ≥ 6 years	Randomised, double blind, placebo-controlled, crossover study, 38 subjects ≥ 6 years	LCI2.5 (LSM ± SE) =	LCI2.5(LSM ± SE) = 0.20 ± 0.19	LSM difference - point estimate: −0.66
	EU Clinical Trials Register (2019)		−0.46 ± 0.19		95%, 2-sided CI: [−1.1; −0.21]
	Specified CFTR gating mutation EU Clinical Trials Register (2018)	Phase 3b, randomised, double blind, placebo-controlled, crossover study with long-term open-label period, 14 subjects	LCI2.5 (AM ± SD) =	LCI2.5 (AM ± SD) =	P-value = 0.2121
			−0.53 ± 1.23	−0.07± 0.93	(Paired t-test)
	R117H-CFTR mutation EU Clinical Trials Register (2015b)	Phase 3, randomised, double blind, placebo-controlled, parallel-group study, 69 subjects	FEV_1_ (LSM ± SE) = 2.57 ± 1.1532	FEV_1_ (LSM ± SE) = 0.46 ± 1.1313	LSM difference - point estimate: 2.1114
					95%, 2-sided CI: [−1.1305; 5.3532]
					P-value = 0.1979
					(MMRM)
	G551D Mutation ≥ 12 years EU Clinical Trials Register (2015a)	Phase 3, Randomized, double-Blind, placebo-controlled, parallel group study, 161 subjects	FEV_1_ (LSM ± SE) = 10.4 ± 0.7	FEV_1_ (LSM ± SE) = −0.2 ± 0.7	LSM difference - point estimate: 10.6
					95%, 2-sided CI: [8.6;12.6]
					*P*-value <0.0001 (α=0.05)
					(MMRM)
	Non-G551D CFTR Gating mutation EU Clinical Trials Register (2015c)	Phase 3, randomised, double blind, placebo-controlled, crossover study with open-label period, 39 subjects	FEV_1_ (LSM ± SE) = 7.49 ± 1.2292	FEV_1_ (LSM ± SE) = −3.19 ± 1.2459	LSM difference - point estimate: 10.6780
					95%, 2-sided CI: [7.2559; 14.1]
					P-value <0.0001
					(MMRM)
Orkambi^®^(lumacaftor/ivacaftor)	Homozygous for F508del CFTR mutation EU Clinical Trials Register (2017c)	Phase 3, randomised, double blind, placebo-controlled, parallel-group study, 206 subjects	LCI2.5 (LSM ± SE) = −1.01 ± 0.13	LCI2.5 (LSM ± SE) = 0.08 ± 0.13	LSM difference - point estimate (SE): −1.09
					95%, 2-sided CI: [−1.43; −0.75]
					*P*-value <0.0001
					(MMRM)
Symkevi^®^ (tezacaftor /ivacaftor)	Homozygous for F508del CFTR mutation EU Clinical Trials Register (2017b)	Phase 3, randomised, double blind, placebo-controlled, parallel-group study, 510 subjects	FEV_1_ (LSM ± SE) = 3.4 ± 0.3	FEV_1_ (LSM ± SE) = −0.6 ± 0.3	LSM difference - point estimate (SE): −1.09
					95%, 2-sided CI: [3.1; 4.8]
					P-value <0.0001
					(MMRM)
	Heterozygous for F508del CFTR mutation and F508del/NR EU Clinical Trials Register (2017a)	Phase 3, randomised, double blind, placebo-controlled, parallel-group study, 168 subjects	FEV_1_ (LSM ± SE) = 1 ± 0.6	FEV_1_ (LSM ± SE) = −0.1± 0.6	LSM difference - point estimate (SE): −1.09
					95%, 2-sided CI: [−0.3; 2.6]
					P-value = 0.1176
					(MMRM)

CFTR, cystic fibrosis transmembrane conductance regulator; LCI, lung clearance index; FEV_1_, Forced expiratory volume in one second; LSM, least square means; SE, standard error; CI, confidence interval; AM, arithmetic mean; SD, standard deviation; MMRM, Mixed model repeated measures; NR, non-responsive to tezacaftor and/or ivacaftor.

Although Orkambi^®^ (lumacaftor/ivacaftor) and Symkevi^®^ have, moderately, while Kalydeco^®^ (ivacaftor) and Kaftrio^®^ (ivacaftor/tezacaftor/elexacaftor) have, greatly, improved quality of life for many patients, access to these medicines is not always guaranteed due to their associated high cost and burden on healthcare budgets (Chevreul et al., 2016; Lopes-Pacheco, 2019). After the adoption of CFTR modulators, a significantly higher expenditure was observed in Europe: a recent study reviewed a database of PWCF and showed that only the four percent of PWCF who were on CFTR modulators caused an increase of 27.5% in CF pharmaceutical spending (Chevreul et al., 2016). This is expected to augment further as the market uptake will grow when all eligible PWCF receive CFTR protein-targeting medicines. Additionally, new CFTR-modulators from Vertex and other companies such as Abbvie and Eloxx Pharmaceuticals are in the pipeline (Cystic Fibrosis Foundation, 2021a; Lopes-Pacheco, 2019). To illustrate, Germany noted an expenditure of €159 million in 2016 and estimates this amount to triple to €594 million if all patients would receive these modulators (Frey et al., 2019).

To inform reimbursement decisions of new medicines, many European jurisdictions perform health technology assessment (HTA) (Morel et al., 2013). For rare disease therapies such as these CFTR modulators, however, high uncertainty on medicine performance exists due to the limited and genetically heterogeneous population as well as adoption of surrogate endpoints in clinical settings (Kent et al., 2014; McLeod et al., 2020). To allow market access of Vertex’ products while accounting for clinical uncertainties and high costs, some healthcare authorities closed mutual agreements with the manufacturer (Morel et al., 2013).

In this study, we aim to comparatively analyze publicly accessible list prices, reimbursement decisions, economic evaluations, budget impact analyses (BIAs), managed entry agreements (MEAs) and multinational collaborations of Kalydeco^®^ (ivacaftor), Orkambi^®^ (lumacaftor/ivacaftor) and Symkevi^®^ (tezacaftor/ivacaftor) in European countries.

## Methods

We conducted a comparative analysis of list prices, reimbursement statuses, economic evaluations, BIAs and MEAs of Kalydeco^®^ (ivacaftor), Orkambi^®^ (lumacaftor/ivacaftor) and Symkevi^®^(tezacaftor/ivacaftor) in selected European countries. Kaftrio^®^ (ivacaftor/tezacaftor/elexacaftor) was not included in the analysis due to limited information availability as it was recently authorized. Twelve countries were selected based on publicly accessible data and consisted of Austria, Belgium, Denmark, France, Germany, Ireland, Poland, Spain, Sweden, Switzerland, the Netherlands, the United Kingdom. If information was confidential or not available for a specific country, the country was not analyzed further.

Official list prices and reimbursement status of the CFTR modulators were recovered from public sources and grey literature, namely, medicinal products databases, formularies and/or pharmaceutical registries and government-specific healthcare or reimbursement databases. The latter comprised of Belgian National Institute for Health and Disability Insurance (NIHDI) and Belgisch Centrum voor Farmacotherapeutische Informatie (BCFI), German Gemeinsamer Bundesausschuss (G-BA) and Rote Liste, Swedish Tandvårds-och läkemedelsförmånsverket (TLV), Scottish Medicines Consortium (SMC), English National Health Service (NHS) and National Institute for Health and Care Excellence (NICE), Dutch Geneesmiddelenvergoedingssysteem (GVS), French Ministère des Affaires Sociale et de la Santé and Centre National Hospitalier d'Information sur le Médicament (CNHIM), Danish Lægemiddelstyrelsen, Austrian Österreichische Sozialversicherung (SV), Irish Health Service Executive (HSE) and Swiss Federal Office of Public Health (FOPH). Additional information on reimbursement status was collected from parliamentary reports and the company’s press releases.

To conduct a comparison between countries, we converted list prices to prices per defined daily dose (DDD) which represents the assumed average maintenance dose per day for a medicine used for its main indication in adults (World Health Organization, 2021). If no DDD of the CFTR modulator was available for a specific dose, instead, we converted list price to price per average daily dose (ADD) as indicated in the package leaflet. For Kalydeco’s^®^ (ivacaftor) dose of 150 mg, a DDD of 0.3g was specified (WHO, 2020). Only for Orkambi’s^®^ (lumacaftor/ivacaftor) tablet dose of 200 mg/125 mg, a DDD of four tablets was stated (WHO, 2021). For the other tablet dose of 100 mg/125 mg of Orkambi^®^ (lumacaftor/ivacaftor), we adopted an ADD of four tablets instead (European Medicines Agency, 2015). For Symkevi^®^ (tezacaftor/ivacaftor), no DDD was released thus the ADD was defined as one 100mg/150 mg Symkevi^®^ (tezacaftor/ivacaftor) tablet combined with one 150 mg Kalydeco^®^ (ivacaftor) tablet. (European Medicines Agency, 2018). List prices comprised of pharmacist fee and value added tax (VAT). If the price was listed without pharmacist fee, it was specified, or without tax, it was recalculated with the VAT rate on prescription-only medicines from the corresponding country (Bundesverband der Pharmazeutischen Industrie (BPI), 2020). Currencies were subsequently converted to 2021 € with Belgium as the target country using the ‘CCEMG - EPPI-Centre Cost Converter’ online tool (The Campbell and Cochrane Economics Methods Group and the Evidence for CCEMG, 2021). It was assumed that original data related to the year of the data source. Finally, prices were rounded to the unit.

To compare economic evaluations, we considered following design parameters; model, perspective, comparator, time horizon, costs and discounting. The incremental cost-effectiveness ratio (ICER) or cost per quality-adjusted life years (QALY) and sensitivity analyses were also included.

Furthermore, publicly available BIAs were reviewed on their design including perspective, time horizon, target population (size), costs, discounting and uncertainty. The results of BIAs were also reported. This information was gathered from reimbursement applications or health technology appraisal reports from the respective agencies.

We determined whether a financial or performance-based MEA, between the company and national healthcare payers for Kalydeco^®^ (ivacaftor), Orkambi^®^ (lumacaftor/ivacaftor) and Symkevi^®^ (tezacaftor/ivacaftor) existed for reimbursement in some European countries. Lastly, the impact of multinational collaborations on market access to PWCF was assessed by reviewing literature. To that end, Pubmed, ISPOR, national healthcare payers’ websites and the company’s official website were consulted.

## Results

### List Prices

**FIGURE 1:**
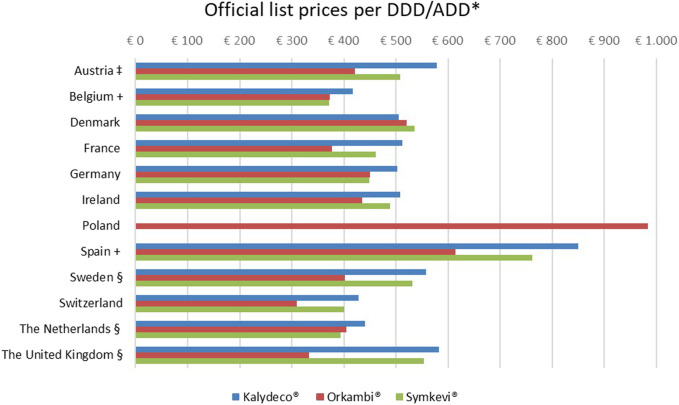
Official list prices per DDD/ADD of Kalydeco^®^, Orkambi^®^ and Symkevi^®^. *DDD*, Defined Daily Dose. *ADD*, Average Daily Dose. *Consulted on September 22, 2021, VAT included and expressed in 2021 €. ^‡^10% VAT rate included manually. ^+^Tablet form of Orkambi^®^: 100/125 mg instead of 200/125 mg ^§^pharmacist fee excluded. References: Austria (Österreichische Sozialversicherung, 2021). Belgium (RIZIV, 2021a; RIZIV, 2021c; RIZIV, 2021b). Denmark (Danish Medicines Agency, 2021c; Danish Medicines Agency, 2021b; Danish Medicines Agency, 2021a). France (Theriaque CNHIM - Centre National Hospitalier d'Information sur le Médicament, 2021a; Theriaque CNHIM - Centre National Hospitalier d'Information sur le Médicament, 2021b; Thériaque CNHIM - Centre National Hospitalier d'Information sur le Médicament, 2021). Germany (Rote Liste, 2021c; Rote Liste, 2021a; Rote Liste, 2021b). Ireland (Health Service Executive (HSE), 2021). Poland (medycyna praktyczna, 2021). Spain (Vademecum, 2021a; Vademecum, 2021c; Vademecum, 2021b). Sweden (Tandvårds-och läkemedelsförmånsverket, 2021c; Tandvårds- och läkemedelsförmånsverket, 2021b; Tandvårds- och läkemedelsförmånsverket, 2021a). Switzerland (Office fédéral de la santé publique, 2021; Open Drug Database, 2021b; Open Drug Database, 2021c; Open Drug Database, 2021a). Netherlands (Zorginstituut Nederland, 2021b; Zorginstituut Nederland, 2021c; Zorginstituut Nederland, 2021d. The United Kingdom (NICE British National Formulary, 2021b; NICE British National Formulary, 2021c; NICE British National Formulary, 2021a).

List prices for Kalydeco^®^ (ivacaftor) ranged from €417 to €850 per DDD in Belgium and Spain, respectively (see Figure 1). For Orkambi^®^ (lumacaftor/ivacaftor), the lowest price was at €309 per DDD in Switzerland and the highest price was at €983 per DDD in Poland. Symkevi^®^ (tezacaftor/ivacaftor) prices varied between €372 per ADD in Belgium and €761 per ADD in Spain. No price for Kalydeco^®^ (ivacaftor) and Symkevi^®^ (tezacaftor/ivacaftor) was available for Poland. Except for in Denmark, Kalydeco^®^ (ivacaftor) was considerably higher priced than Orkambi^®^ (lumacaftor/ivacaftor) and Symkevi^®^ (tezacaftor/ivacaftor). Compared to Orkambi^®^ (lumacaftor/ivacaftor), Symkevi^®^ (tezacaftor/ivacaftor) was more expensive apart from in Belgium, Demark, Germany and France where both treatments’ prices were similar.

### Reimbursement Status

The three CFTR modulators were fully reimbursed in ten out of 12 examined countries except from Sweden and Poland (see Table 2). In France, healthcare authorities decided to officially reimburse Kalydeco^®^ (ivacaftor), Orkambi^®^ (lumacaftor/ivacaftor) and Symkevi^®^ (tezacaftor/ivacaftor) at a partial rate of 65%, however, PLWCF were exempt from any out-of-pocket costs through the long-lasting illness (ALD) scheme (Haute Autorité de Santé, 2014, Haute Autorité de Santé, 2019; Haute Autorité de Santé, 2020c, APM News, 2021; L’assurance maladie (ameli), 2021; Vaincre la mucoviscidose, 2021). A reimbursement decision for Symkevi^®^ (tezacaftor/ivacaftor) was reached in 2021, 1 year after the positive reimbursement advice in May of 2020 (Haute Autorité de Santé HAS, 2020c; Vertex Pharmaceuticals Incorporated, 2021). Furthermore, in Switzerland, Kalydeco^®^ (ivacaftor) reimbursement is conditioned by particular clinical modifications (Bundesamt für Gesundheit BAG, 2015). The Swedish TLV negatively decided on reimbursement of Kalydeco^®^ (ivacaftor) and Symkevi^®^ (tezacaftor/ivacaftor) (Tandvårds-och läkemedelsförmånsverket TLV, 2019). In Poland, no CFTR modulator is currently reimbursed and an official administrative decision after negative advice from the Economic Commission is awaited (Miłkowski, 2020; Munyama et al., 2020).

**TABLE 2:** Reimbursement status of Kalydeco^®^, Orkambi^®^ and Symkevi^®^ in specific European countries.^a^

CFTR modulator	Country^b^														
	Austria	Belgium	Denmark	England	France	Germany	Ireland	Northern Ireland	Poland	Scotland	Spain	Sweden	Switzerland	The Netherlands	Wales
**Kalydeco®**	**✔**	**✔**	**✔**	**✔**	**✔**	**✔**	**✔**	**✔**	**✖**	**✔**	**✔**	**✖**	**✔**	**✔**	**✔**
**Orkambi®**	**✔**	**✔**	**✔**	**✔**	**✔**	**✔**	**✔**	**✔**	**✖**	**✔**	**✔**	**✔**	**✔**	**✔**	**✔**
**Symkevi®**	**✔**	**✔**	**✔**	**✔**	**✔**	**✔**	**✔**	**✔**	**✖**	**✔**	**✔**	**✖**	**✔**	**✔**	**✔**

a As of June 2021.

b References in order of the listed countries: (Österreichische Sozialversicherung, 2020), (Comissie voor Gezondheid en Gelijke Kansen, 2019; RIZIV, 2020), (ISPOR, 2015; Nawrat, 2018), (National Health Service (NHS) England, 2019; NHS Derbyshire Medicines Management and Clinical Policies, 2020), (Haute Autorité de Santé HAS, 2020a; Haute Autorité de Sant é , 2020b), (Mukoviszidose eV, 2012; Mukoviszidose, 2018a; Mukoviszidose, 2018b; Gehl, 2021), (Health Service Executive (HSE), 2020; HSE PCRS, 2020), (Vertex Pharmaceuticals Incorporated, 2019c), (Miłkowski, 2020; Munyama et al., 2020), (Cystic Fibrosis Trust, 2016; Vertex Pharmaceuticals Incorporated, 2019b), (Vademecum, 2020a; Vademecum, 2020b; Vademecum, 2020c), (Vertex Pharmaceuticals Incorporated, 2018b), (Office fédéral de la santé publique, 2020), (Zorginstituut Nederland, 2020b; Zorginstituut Nederland, 2020c; Zorginstituut Nederland, 2020e), (Vertex Pharmaceuticals Incorporated, 2019d).

✔, full reimbursement; ×, no reimbursement (full out-of-pocket expenses).

### Economic Evaluations

Tables 3 – 5 show the company’s and/or health authorities’ economic evaluations per investigated country, in terms of design and cost-effectiveness (ICER) for Kalydeco^®^ (ivacaftor), Orkambi^®^ (lumacaftor/ivacaftor) and Symkevi^®^ (tezacaftor/ivacaftor), respectively.

**TABLE 3:** Overview of the economic evaluation of different European countries for Kalydeco^®^ (ivacaftor).^a^

Country	Indication	Design						Currency	ICER(€; 2020 values)^b^	Sensitivity analyses
		model	Reference year	comparison	time horizon	Costs	discounting			
**Belgium**	Gating class III	Exemption of economic evaluation for orphan medicines								
	≥ 2 years									
**England** Whiting et al. (2014)	G551D ≥ 6 years	Patient-level micro-simulation	Payer	SoC vs SoC + ivacaftor	Lifetime	Medicine acquisition, treatment directly related to CF, lung transplantation	Discount rate of 3.5%	£ 2014	£771,297/QALY **(1,001,336)**	Deterministic (optimistic-conservative scenario):
										334,775/QALY - 1,273,805/QALY
										PSA:
										607,699/QALY -
										1,047,179/QALY
										mean: 814,401/QALY
										Most sensitive to long-term effectiveness (ppFEV_1_, weight, exacerbations) and long-term costs of ivacaftor
**France**	G551D	Confidential								
	≥ 6 years									
	Gating class III									
	≥ 6 years									
**Germany**	Gating class III	Confidential								
**Ireland** (National Centre for Pharmacoeconomics (NCPE), 2013a **;** NCPE, 2016a **;** NCPE, 2017a)	G551D	Patient-level micro-simulation	Payer	SoC vs SoC + ivacaftor	Lifetime	Disaggregated costs were difficult to assess	Not reported	€ 2013	449,035/QALY (**500,195)**	Deterministic (optimistic - conservative scenario): 500,195/QALY – 855,437 /QALY
	≥ 6 years									Most sensitive to ppFEV_1_
	Gating class III ≥ 2 years			A. SoC vs early ivacaftor (initiated at two years of age) + SoC B. Early ivacaftor + SoC vs late ivacaftor (initiated at six years of age) + SoC		Not reported	Discount rate of 5%	€ 2016	A. 465,546/	Price of ivacaftor would have to fall below 25,000/QALY per patient per annum to bring the ICER close threshold
									QALY	
									(**487,122)**	
									B. 636,237/	
									QALY	
									**(665,723)**	
	R117H ≥ 18 years			SoC vs SoC + ivacaftor		Hospitalization,	Discount rate of 5%	€ 2017	444,466/QALY **(463,288)**	Most sensitive to discount rates, adherence to ivacaftor and mean absolute change in ppFEV_1_ Price of ivacaftor would have to fall to 34,692/QALY to give an ICER of 45,000/QALY i.e. a 6.7-fold price reduction
						lung transplantation,				
						medicine acquisition including SoC (mucolytics, pancreatic enzymes, beta agonists and				
						antibiotics)				
**Poland** Agencja Oceny Technologii Medycznych i Taryfikacji (2015); MAHTA, (2019)	Gating class III ≥ 12 months old	Patient-level micro-simulation	Payer; Societal	SoC vs SoC + ivacaftor	Lifetime	Direct medical costs: Medicine acquisition,qualification and treatment monitoring, standard of care, exacerbation treatment, adverse events, lung transplantation Indirect costs: loss of productivity due to absenteeism, care, premature death	discount rate in base case analysis of; 5% for costs, 3.5% for health outcomes	NA	Confidential	No details on the scenario analysis and PSA performed
**Scotland** (SMC), 2013; SMC, 2016a; SMC, 2016b)	G551D ≥ 6 years	Patient-level micro-simulation	Payer	SoC vs SoC + ivacaftor	Lifetime	all hospital and community care, treatment	Not reported	£ 2013	277,011/QALY **(365,782)**	Univariate:
										266,364/QALY -321,904/QALY
										Scenario:
										373,964/QALY -
										562,617 /QALY
										Most sensitive to ppFEV_1_ and age
										Results include agreed refund under Patient Access Scheme
	Gating class III ≥ 2 years			A. SoC vs early ivacaftor treatment (initiated at two years of age) + SoC		Medicines,disease management,hospitalization,lung transplantation	Discount rate of 1.5%	£ 2016	A. 609,316/QALY	Deterministic:
				B. Early ivacaftor treatment + SoC vs late ivacaftor treatment (initiated at six years of age) + SoC			in base case analysis and sensitivityAnalysis		**(771,711)**B. 484,386/QALY **(613,485)**	A. 625,272/QALY -2,369,999 /QALYB. 470,051/QALY -1,023,073/QALYSensitive to discount rate, utility values and treatment efficacy
				
				
				
				
				
				
	R117H ≥ 18 years			SoC vs SoC + ivacaftor		Medicine acquisition, disease management, lung transplantation,adverse events	Discount rate of 3.5%	£ 2016	473,071/QALY **(599,154)**	Univariate:
										490,062/QALY -
										880,326/QALY
										Scenario:
										208,254/QALY - 621,562/QALY
										Sensitive to the discount rate, utility values and treatment efficacy (ppFEV_1_, exacerbations).
**Sweden** Tandvårds- och läkemedelsförmånsverket TLV (2014); Tandvå rds- och l ä kemedelsf ö rm å nsverket (2018b))	G551D ≥ 6 years	Patient-level micro-simulation	Payer (company); Societal (NHS)	SoC vs SoC + ivacaftor	Lifetime	Medicine acquisition	Discount rate of 3%	SEK 2014	Company:	Company: 4,755,152/QALY - 7, 282,842/QALY
									3,474,120/QALY **(361,444)**	
									NHS:	
									5,840,000/QALY (**607,588)**	
									- 10,440,000/QALY	
									**(1,086,167)**	
	Gating class III ≥ 2 years					Direct costs:Medicine acquisition, healthcare and resource utilization: medicine follow-up, disease management, lung transplantation	Discount rate of 3%	SEK 2018	NHS:	Optimistic - conservative scenario: 4,200,000/QALY - 7,005,198/QALY Most sensitive to disease progression: lung capacity, exacerbations, survival
									5,556,831/QALY	
									**(533,034)**	
									- 7,005,198/QALY	

									**(671,968)**	
									depending on	
									treatment adherence	
**The Netherlands** Zorginstituut Nederland (2014)	Gating class III ≥ 2 years	Markov (company) Patient-level micro-simulation (Health Insurance Funds)	Payer (company); Societal (NHS)	SoC vs SoC + ivacaftor	Lifetime	Direct medical costs: outpatient visits, hospitalizations, medicine acquisition, pharmacy	Yes (NHS:) Costs discounted with 4% Treatment effects discounted with 1.5%	€ 2014	Company: 172,278/QALY (**191,910)** Health Insurance Funds: 266,074/QALY **(296,395)**	Company: 148,000/QALY - 500,023/QALY PSA: mean: 174,945/QALY Health Insurance Funds: 175,291/QALY - 289,476/QALY Most sensitive to drug costs, survival rates and disease progression (ppFEV_1_) and discount rates 0% chance that ivacaftor is cost effective with threshold of 80,000/QALY
**Wales** All Wales Therapeutics and Toxicology Centre (2013, 2015) **;** All Wales Therapeutics and Toxicology Centre, (2017)	G551D≥ 6 years	Patient-level micro-simulation	Not reported	SoC vs SoC + ivacaftor	Lifetime	Medicine acquisition (includes discount of Wales Patient Access Scheme)	Discount rate of 3.5% for costs	NA	Confidential ICER for ivacaftor exceeds conventional thresholds of cost-effectiveness	Sensitivity and scenario
										analyses demonstrate that ICERs greater than that reported in the base case analysis
										may be plausible.
										Sensitive to lung function FEV_1_, utilities and generic price assumptions
	Gating class III ≥ 2 years	Patient-level micro-simulation	Not reported	SoC vs SoC + ivacaftor	Lifetime	Medicine acquisition (includes discount of Wales Patient Access Scheme)	Discount rate of 3.5% for costs	NA	Confidential	One-way scenario analyses: Sensitive to lung function ppFEV_1_
	R117H ≥ 18 years	Patient-level micro-simulation	Payer	SoC vs SoC + ivacaftor	Lifetime	Medicine acquisition (includes discount of Wales Patient Access Scheme), hospitalization, adverse events, transplantation	Discount rate of 3.5% for costs	NA	Confidential ICER is most sensitive to discount rates, the utility equation and mean absolute change in ppFEV_1_	Probabilistic sensitivity analysis suggests that the model results are robust.Probability of ivacaftor to be cost-effective at the willingness-to-pay thresholds of 20,000/QALY and 30,000 is 0%.

CF, cystic fibrosis; ICER, incremental cost-effectiveness ratio; NA, not applicable, NHS, National Health Service; ppFEV_1_, per cent predicted forced expiratory volume in one second; PSA, probabilistic sensitivity analysis; QALY, quality adjusted life years; SoC, standard of care.

a As of June 2021.

b ICER in regular font indicates the results in the currency and reference year used in the study (third column from the right). The number in bold indicates the ICER in euros (Belgium, 2020 values).

**TABLE 4:** Overview of the economic evaluation of different European countries for Orkambi^®^ (lumacaftor/ivacaftor).^a^

Country	Design						Currency Reference year	ICER (€; 2020 values)^b^	ICER (sensitivity analyses)
	model	perspective	comparison	time horizon	costs	discounting			
**Belgium** Zorginstituut Nederland (2016b)	Patient-level micro-simulation	Payer	SoC vs SoC + ivacaftor/lumacaftor	Lifetime	Direct medical costs: medicine acquisition, disease management, exacerbations, hospitalization	Discount of 3% on future costs; 1.5% on future effects	€ 2016	453,286/QALY**(482,727)**	Deterministic:
									311,979/QALY - 1,086,480 /QALY
									Most sensitive to discount rates, medicine costs, decline FEV_1_, utility values
									PSA: 434,370/QALY
									Chances of lumacaftor/ivacaftor being cost-effective is 0%.
**England** NICE (2015)	Patient-level micro-simulation	NHS payer; personal social services payer	SoC vs SoC + ivacaftor/ lumacaftor	Lifetime	Direct medical costs: Management, hospitalization, medicine acquisition, lung transplantation, adverse events	Discount rate of 3.5% on costs and health outcomes	£ 2015	Company: 218,248/QALY **(282,112)** NHS: 221,992/QALY **(286,951)**	Company:
									160,000/QALY – 280,000/QALY
									PSA: mean of 214,838/QALY
									Most sensitive to rate of ppFEV_1_ decline and discount rate, disease management costs/utility values
									NHS:
									197,790**/**QALY - 349,337/QALY
									0% chance of being cost-effective at thresholds of 30,000/QALY and 50,000/QALY
**France** HAS Service évaluation économique et santé publique (2016)	Patient-level micro-simulation	Third party payer	SoC vs SoC + ivacaftor/ lumacaftor	Lifetime	Direct medical costs: medicine acquisition, management, exacerbation, hospitalization, follow-up, transplantation, liver tests	Not reported	€ 2016	622 131/QALY **(675,947)**	Optimistic - pessimistic scenario:
									574 390/QALY - 1,286,625/QALY
									PSA:
									(optimistic) 90% of being more cost effective if willingness-to-pay is 632 000/QALY
									(intermediate) 90% of being more cost effective if willingness-to-pay is 684 000/QALY
									Most sensitive to medicine acquisition costs, age, time horizon, FEV_1_% decline, adherence rate
**Germany**	Confidential								
**Ireland** (National Centre for Pharmacoeconomics NCPE (2016b)	Patient-level micro-simulation	Payer	SoC vs SoC + ivacaftor/ lumacaftor	Lifetime	Direct medical costs:medicine acquisition, pulmonary exacerbation, lung transplantation	Yes, but rate not reported	€ 2016	Company:	Company:
								369,141/QALY	PSA: 370,754/QALY
								**(386,249)**	Probability of being cost-effective is 0%.
								NHS:	Most sensitive to decline rate, discount rate, medicine acquisition cost
								649,624/QALY	
								**(679,731)**	
**Poland ** (Agencja Oceny Technologii Medycznych i Taryfikacji (AOTMIT), 2018; MAHTA, (2019))	Patient-level micro-simulation	Payer; Societal	SoC vs SoC + ivacaftor/ lumacaftor	Lifetime	Direct medical costs: Medicine acquisition, standard of care, exacerbation, lung transplantation, adverse event, monitoringIndirect costs (societal perspective): productivity loss, informal care for children and death	discount rate of 5% for costs and 3.5% for health outcomes	NA	Confidential	Confidential
**Scotland** (Scottish Medicines Consortium (SMC), 2019)	Patient-level micro-simulation	Payer; Societal	SoC vs SoC + ivacaftor/ lumacaftor	Lifetime	Direct costs: Medicine acquisition, disease management	Not reported	£ 2019	214,772/QALY **(256,486)**	Deterministic: 183,037/QALY - 236,034/QALY most sensitive to discount rates for cost and benefits, treatment utility increment, treatment compliance rates, utility values stratified by ppFEV_1_
**Sweden **(Tandvårds- och läkemedelsförmånsverket TLV, 2018a)	Patient-level micro-simulation	Societal	SoC vs SoC + ivacaftor/ lumacaftor	Lifetime	Direct medical costs:Medicine acquisition, lung transplantation, adverse event, hospitalization, follow-up Indirect costs: loss of production (not included in NHS analysis)	Discount rate of 3%	SEK 2018	Company:	NHS:
								Confidential	Univariate: 1,414,988**/**QALY - 1,865,827/QALY
								NHS:	Sensitive to changes in medicine acquisition, age, duration of treatment and useful weights.
								1,541,295/QALY **(147,723)**	Results include agreed refund between company and NHS
								**-** 1,650,000/QALY **(158,275)**	
								depending on total patient number	
**The Netherlands **(Zorginstituut Nederland, 2016b)	Patient-level micro-simulation	Societal	SoC vs SoC + ivacaftor/lumacaftor	Lifetime	Medicine acquisition, Direct medical costs, direct non-medical costs, indirect non-medical costs	Discount rate of 4% on costs and 1.5% on health outcomes	€ 2016	402,883/QALY **(443,330)**	Univariate: 277,288/QALY - 965,668/QALY
									Scenario:
									274,920/QALY - 41,659,132/QALY
									Most sensitive to discount rates, medicine acquisition costs, decline FEV_1_, utility values
									Chances of lumacaftor/ivacaftor being cost-effective is 0%. The price of lumacaftor/ivacaftor should decrease with 82% to be cost-effective.

a As of June 2021.

b ICER in regular font indicates the results in the currency and reference year used in the study (third column from the right). The number in bold indicates the ICER in euros (Belgium, 2020 values).

CF, cystic fibrosis; ICER, incremental cost-effectiveness ratio; NA, not applicable; NHS, National Health Service; ppFEV_1_, per cent predicted forced expiratory volume in one second; PSA, probabilistic sensitivity analysis; QALY, quality adjusted life years; SoC, standard of care.

**TABLE 5:** Overview of the economic evaluation of different European countries for Symkevi^®^ (tezacaftor/ivacaftor).^a^

Country	Indication	Design						Currency Reference year	ICER (€; 2020 values)^b^	ICER (sensitivity analyses)
		model	perspective	comparison	time horizon	Costs	discounting			
**Belgium**	Not conducted									
**England **(NICE, 2018b; NICE, 2018a)	homozygous for the F508del mutation	NA	NHS payer; personal social services payer	SoC vs SoC + ivacaftor/tezacaftor	Long enough, but not specified	NA	NA	NA	Appraisal was halted	
	heterozygous for the F508del mutation and aresidual function mutation									
**France **(HAS Service évaluation économique et santé publique, 2020)	heterozygous for the F508del mutation and a residual function mutation ≥12 years: P67L, R117C, L206W, R352Q, A455E, D579G, 711+3A→G, S945L, S977F, R1070W, D1152H, 2789+5G→A, 3272-26A→G, et 3849+10kbC→T	Patient-level micro-simulation	Third-party payer	SoC vs SoC + ivacaftor/ tezacaftor in combination therapy with ivacaftor	Lifetime	Direct medical costs: medicine acquisition, follow-up, maintenance, pulmonary exacerbation, hospitalization, home care, decline of ppFEV1, adverse event	Discount rate of 4%	€ 2020	Company:	Deterministic:
									945,278/QALY (**980,110)**	745,813/QALY – 1,170,499/QALY
									NHS: **>**1,000,000/QALY	most sensitive to utility, treatment effects ppFEV_1_ and pulmonary exacerbation ratio and medicine cost
									**(>1,036,849)**	Scenario:
										ICER up to 1,402,820/QALY
										Most sensitive to time horizon, discount rates, utility values,
										PSA: willingness-to-pay at 1,100,000/QALY for Symkevi^®^ to have 84% probability of being cost-effective
**Germany **(IHS Global, 2013; Gerber-Grote et al., 2014)	Economic evaluations are not considered									
**Ireland**	Not conducted									
**Poland**	Not conducted									
**Scotland **(Scottish Medicines Consortium, 2019)	homozygous for the F508del mutation ≥ 12 years	Patient-level micro-simulation	Payer; Societal	SoC vs SoC + ivacaftor/ tezacaftor	Lifetime	Medicine acquisition, monitoring, adverse events, disease management, lung transplantation	Yes, but rate not reported	£ 2019	421,173/QALY **(502,974)**	Univariate:
										310,000/QALY to 730,000/QALY
										most sensitive to treatment compliance rate, baseline utility scores, treatment-specific utility increase, decline in ppFEV_1_ and pulmonary exacerbations rate
										Scenarios: ICER did not drop below 300,000/QALY
	heterozygous for the F508del mutation and a residual function mutation							£ 2019	360,499/QALY **(430,516)**	Univariate:
										240,000**/**QALY - 720,000/QALY
										most sensitive to treatment compliance rate, baseline utility scores, treatment-specific utility increase, decline in ppFEV_1_ and pulmonary exacerbations rate
											Scenarios: ICER did not drop below 245,000/QALY
**Sweden **(Tandvårds- och läkemedelsförmånsverket TLV, 2018b)	homozygous for the F508del mutation	Patient-level micro-simulation	Payer	SoC + ivacaftor/ tezacaftorvs SoC + lumacaftor	Lifetime	Direct medical costs: medicine acquisition, follow-up, management, adverse event, care, resource consumption, lung transplantation	Discount rate of 3%	NA	Company and NHS: Confidential	The cost of medicines for Symkevi^®^ + Kalydeco^®^ at €179,057 is higher than the medicine cost for Orkambi^®^ at €145,803.
	heterozygous for the F508del mutation and a residual function mutation						SoC vs SoC + ivacaftor/ tezacaftor	SEK 2018	NHS: 5 675 522/QALY **(544,420)** – 7 035 099/QALY **(674,836)** depending on treatment adherence	NHS:
										2,863,762/QALY – 9,220,377/QALY
											Most sensitive to treatment compliance, time horizon and treatment interruption
**The Netherlands **(Zorginstituut Nederland, 2020d)	heterozygous for the F508del mutation and a residual function mutation (no cost-effectiveness of homozygous mutation)	Patient-level micro-simulation	Societal	SoC vs SoC + ivacaftor/ tezacaftor in combination therapy with ivacaftor	Lifetime	Direct-medical costs: medicine acquisition, adverse event, lung transplantation, monitoring, hospitalization, exacerbation Indirect medical costs: patient and family, productivity	Discount of 4% on future costs and discount of 1,5% on future effects	€ 2020	376,060/QALY **(387,456)**	Univariate:
										most sensitive to decline in ppFEV1 followed by, utility of ppFEV_1_, PEx rate ratio and compliance
										Scenario: up to 600,421/QALY
										PSA: 378,010/QALY
										Chances of Symkevi^®^ being cost effective is 0% with threshold of €80,000.
										Price should decrease with 80%

a As of June 2021.

b ICER in regular font indicates the results in the currency and reference year used in the study (third column from the right). The number in bold indicates the ICER in euros (Belgium, 2020 values).

CF, cystic fibrosis; ICER, incremental cost-effectiveness ratio; NA, not applicable; NHS, National Health Service; PEx, pulmonary exacerbation; ppFEV1, per cent predicted forced expiratory volume in one second; PSA, probabilistic sensitivity analysis; QALY, quality adjusted life years; SoC, standard of care.

#### Design

For Kalydeco^®^ (ivacaftor) an economic evaluation was provided by the company and/or the country-specific healthcare authorities in the indications of G551D in children above six, gating class III children above two and/or R117H mutations in adults over 18 (see Table 3). A patient-level micro-simulation model, payer perspective, ivacaftor plus standard of care with standard of care only comparison and a lifetime horizon were adopted in most countries. However, differences were detected for: the Netherlands where the company carried out an evaluation based on a Markov model and the health authorities adopted a societal perspective; Poland and Sweden for which, respectively, the company and health authority adopted a societal perspective next to the payer’s perspective; Scotland and Sweden, for which early ivacaftor treatment (initiated at 2 years of age) was additionally compared to standard of care and late ivacaftor treatment (initiated at 6 years of age); Wales, for which the perspective in G551D and gating class III mutations indications were not reported. Adjustments to the economic evaluation design made by local health authorities were claimed to be more adaptive to their population’s characteristics. Reported costs often included medicine costs but also direct condition-related costs and indirect non-medical costs assessed in Poland. Furthermore, a discount rate for costs and/or health outcomes and sensitivity analyses, scenario or probabilistic, were generally considered.

In all countries, Orkambi^®^ (lumacaftor/ivacaftor) was evaluated for people homozygous for the F508del *CFTR* mutation (see Table 4). In every economic evaluation, a patient-level micro-simulation model, third-party payer perspective and/or societal perspective was adopted. The treatment combined with the standard of care was compared to standard of care only. A lifetime horizon and medicine costs but also direct medical costs were generally considered. Poland and the Netherlands were the sole countries to also include indirect costs in their evaluation. When reported, a discount rate was applied to costs and health outcomes.

Symkevi^®^ (tezacaftor/ivacaftor) was evaluated in its indication either in people homozygous for the F508del mutation and/or heterozygous for the F508del mutation with residual function mutation (see Table 5). Again, third-party payer perspective and/or societal perspective was adopted while the intervention, tezacaftor/ivacaftor combination therapy with ivacaftor, plus standard of care was compared to either standard of care only or standard of care and lumacaftor in the case of Sweden. Lifetime was generally considered as a time horizon. Furthermore, medicine costs, direct medical costs, and additionally, for the Netherlands, indirect costs were integrated in the economic evaluations. A discount rate on costs and/or outcomes was also applied.

#### Cost-Effectiveness (Incremental Cost-Effectiveness Ratio)

##### Kalydeco^®^ (Ivacaftor)

The ICERs varied greatly per indication and across the analyzed countries (see Table 3). An ICER of €1M per QALY in the G551D indication was predicted for England. The company provided ICERs for G551D, gating class III (initiated at two or 6 years of age) and R117H mutations: for Ireland these were, respectively, €500K per QALY, €487K per QALY or €666K per QALY and €463K per QALY whereas for Scotland, values were, respectively, €366K per QALY, €772K per QALY or €613K per QALY and €599K per QALY. For Sweden, in the indication of G551D, the company estimated an ICER of €361K per QALY whereas their health authority adjusted this value to an ICER ranging between €608K and €1.1M per QALY and reported an ICER between €533K and €672K per QALY in the gating class III indication. Likewise, for gating class III mutations in the Netherlands, the company provided an ICER of €192K per QALY that was adjusted by their health insurance fund to a value of €296K per QALY. The ICER values predicted by local health authorities were generally higher, more accurate and less varying. Both deterministic and probabilistic sensitivity analyses showed that ICERs were, generally, most sensitive to treatment efficacy measurements, costs of ivacaftor, discount rates, age and utility values. France, Poland and Wales did not publicly disclose their ICER estimations.

##### Orkambi^®^ (Lumacaftor/Ivacaftor)

The company predicted an ICER of €483K per QALY for Belgium, €282K per QALY for England, €676K per QALY for France, €386K per QALY for Ireland, €256K per QALY for Scotland and €443K per QALY for the Netherlands (see Table 4). English and Irish health authorities corrected the company’s predicted ICER to €287K per QALY and €680K per QALY, respectively. The Swedish health authority predicted an ICER between €148K and €158K per QALY while the company’s ICER was confidential. The ICERs, after correction by health authorities were estimated to be significantly higher than the company’s predictions. For England and Ireland, this meant an ICER that was approximately €5,000 per QALY and €300,000 per QALY, respectively, higher than the company’s predicted ICERs. For countries like England, Ireland and the Netherlands, that rely on an ICER threshold for reference, Orkambi^®^ (lumacaftor/ivacaftor) had zero percent chances of being cost-effective. In France, Orkambi^®^ (lumacaftor/ivacaftor) had a 90% probability of being cost-effective if the willingness-to-pay would at least be €632K per QALY while the Netherlands reported that the price of Orkambi^®^ (lumacaftor/ivacaftor) should be reduced with about 82% to be deemed cost-effective. Deterministic and probabilistic sensitivity analyses indicated that ICER values were most sensitive to medicine costs, age, time horizon, treatment outcomes, adherence, utility values and discounting. No cost-effectiveness estimate was publicly available for Poland.

##### Symkevi^®^ (Tezacaftor/Ivacaftor)

The company reports an ICER for France of €980K per QALY in the heterozygous indication whereas, an ICER of €503K per QALY for homozygotes and of €431K per QALY for heterozygotes was predicted for Scotland (see Table 5). For the Netherlands, an ICER of €387K per QALY in the heterozygous indication was estimated. However, health authorities in France believed the ICER prediction of the company to be an underestimation and claimed a more accurate ICER, specific to its population characteristics, to be above €1M per QALY. For Sweden, only an ICER value estimated to be between €544K and €675K per QALY for heterozygotes was reported by their health authority. Moreover, for homozygotes, Symkevi^®^ (tezacaftor/ivacaftor) treatment cost was valued to be approximately €34,000 more expensive than that of Orkambi^®^ (lumacaftor/ivacaftor). ICER ranges estimated by the health authorities were generally higher and depicted a smaller variation between the values. For all listed ICERs, a deterministic analysis was performed which showed highest sensitivity for utility values, treatment effects, medicine costs, adherence and discount rates. Probabilistic sensitivity analysis for France showed that the willingness-to-pay should be set at €1.1M for Symkevi^®^ (tezacaftor/ivacaftor) to have an 84% probability of being cost-effective. For the Netherlands a price decrease of 80% would be required as Symkevi^®^ (tezacaftor/ivacaftor) would have zero percent chances of being cost-effective considering the current price and threshold. England, Germany, Ireland and Poland had no public data on cost-effectiveness available.

#### Country-Specific Outcomes

Belgium does not consider cost-effectiveness for orphan medicines (Denis et al., 2009). In their analysis for Orkambi^®^ (lumacaftor/ivacaftor), jointly assessed with the Netherlands, the Dutch ICER threshold of €80,000 per QALY was used as a reference and Orkambi^®^ (lumacaftor/ivacaftor) was deemed not cost-effective.

Data on economic evaluations by the French HTA Agency (CEESP) were available for Orkambi^®^ (lumacaftor/ivacaftor) and Symkevi^®^ (tezacaftor/ivacaftor), although France generally does not consider cost-effectiveness for reimbursement (Denis et al., 2009; Haute Autorité de Santé HAS, 2014; Haute Autorité de Santé, 2019; Haute Autorité de Santé, 2020c). For Orkambi^®^ (lumacaftor/ivacaftor) and Symkevi^®^ (tezacaftor/ivacaftor), CEESP reported significant clinical uncertainties considering long term efficacy on FEV_1_% and pulmonary exacerbations and required a significant reduction in price for the interventions to be deemed cost-effective.

The Dutch Healthcare Institute adopts a threshold to determine cost-effectiveness and inform their Health Minister on reimbursement (Denis et al., 2009). The price of Kalydeco^®^ (ivacaftor) would have to be reduced by 82% for the treatment to bring the ICER below the thresholds and be deemed cost-effective. Orkambi^®^ (lumacaftor/ivacaftor) was given negative advice for reimbursement due to failed cost-effectiveness, insufficient clinically proven effect, lack of long-term data on lung function but also a limited patient eligibility (Zorginstituut Nederland, 2016a; Zorginstituut Nederland, 2019a). Symkevi^®^ (tezacaftor/ivacaftor) was negatively advised for heterozygotes but positively advised for homozygotes with the condition that the price would not be set higher than Orkambi^®^ (lumacaftor/ivacaftor)’s price given that it has a similar therapeutic value (Zorginstituut Nederland, 2019b; Zorginstituut Nederland, 2020a).

In Sweden, cost-effectiveness is flexible, influenced by disease severity and usually determined based on a range of €35,000 to €100,000 per QALY (Denis et al., 2009). However, cost-effectiveness is not a primary criterium and no official threshold is defined. Kalydeco^®^ (ivacaftor)’s and Symkevi^®^ (tezacaftor/ivacaftor)’s costs were not deemed reasonable compared to their clinical benefit and therefore not funded for any of their indication (Tandvårds-och läkemedelsförmånsverket TLV, 2019). In contrast, Orkambi^®^ (lumacaftor/ivacaftor) was funded with the requirement to register specific effect parameters and a reduced cost.

For England, Kalydeco^®^ (ivacaftor) was shown not to be cost-effective unless a discount would be agreed and ICER would fall within the increased ultra-orphan medicines threshold margin of £100,000 to £300,000 per QALY (Whiting et al., 2014; NHS England, 2015; National Institute for Health and Care Excellence (NICE), 2017; Kelly et al., 2018). Orkambi^®^ (lumacaftor/ivacaftor) had zero percent chance of being cost-effective compared to the standard of care at their official threshold of £30,000 per QALY and was given a negative recommendation.

The Scottish Medicines Consortium (SMC) does not specify a formal ICER cut off but NHS’ threshold of £20,000 per QALY is often used as a reference (Scottish Medicines Consortium, 2021a). In some cases, a higher cost per QALY may be accepted and additional factors are assessed to determine value for money (Denis et al., 2009). SMC did not advise Kalydeco^®^ (ivacaftor), Orkambi^®^ (lumacaftor/ivacaftor) nor Symkevi^®^ (tezacaftor/ivacaftor) for reimbursement within NHS Scotland because of insufficient justification of the cost in relation to the health benefit and a lack of robust economic and clinical analysis (Scottish Medicines Consortium (SMC), 2013; SMC, 2016a; SMC, 2016b; Kelly et al., 2018; Scottish Medicines Consortium, 2019).

Ireland considered incremental cost-effectiveness with a threshold of €45,000 per QALY in their economic evaluation (National Centre for Pharmacoeconomics (NCPE), 2017b). NCPE suggested significant price reductions for Kalydeco^®^ (ivacaftor) and Orkambi^®^ (lumacaftor/ivacaftor) as acquisition costs were very high; no cost-effectiveness was proven and long-term clinical data was absent (National Centre for Pharmacoeconomics (NCPE), 2013b; NCPE, 2016c). Symkevi^®^ (tezacaftor/ivacaftor) was not subject to HTA.

In Poland, cost-effectiveness with an ICER threshold of three times their GDP per capita of that year, is considered (Kolasa et al., 2018). The Polish HTA Agency (AOTMiT) gave a negative recommendation for Kalydeco^®^ (ivacaftor) and Orkambi^®^ (lumacaftor/ivacaftor) because of insufficient clinical evidence, poor quality data and cost in relation to the benefit being insufficiently justified (Agencja Oceny Technologii Medycznych i Taryfikacji, 2015; Agencja Oceny Technologii Medycznych i Taryfikacji (AOTMIT), 2018). No HTA report for was currently available for Symkevi^®^ (tezacaftor/ivacaftor) although a reimbursement application was filed in February of 2021 (Oddech Zycia, 2021).

In Wales, the English NHS threshold of £100,000 to £300,000 per QALY for the economic evaluation of ultra-orphan medicines was adopted (Denis et al., 2009; National Institute for Health and Care Excellence (NICE), 2017). Kalydeco^®^ (ivacaftor) was negatively recommended by their health technology assessment body (AWMSGs) as issues surrounding cost-effectiveness and clinical uncertainties were defined (Drakeford, 2013; All Wales Medicines Strategy Group (AWMSG), 2019).

#### Budget Impact Analyses

##### Kalydeco^®^ (Ivacaftor)

For most countries a BIA was provided in the indication of gating class III mutations and/or G551D mutations (see Table 6). In addition, England, Scotland and Wales also analyzed the budget impact in the indication of R117H mutations. In terms of design, payer’s perspective was adopted in all cases, except for Wales that did not report their perspective. Budget impact results were depicted over an annual, 3-year, 5-year and/or life time horizon. Population size varied per country and depending on the indication. An open population was considered in Ireland, Scotland and Wales, in the indications G551D and R117H mutations. In Scotland, market uptake of Kalydeco^®^ (ivacaftor) for the gating class III and for G551D mutations were estimated to be 100 and 90%, respectively. For the Netherlands a market expansion with a treatment uptake of 100% was predicted. Medicine only costs were considered in Belgium, Poland, Sweden and the Netherlands, disaggregated costs were not reported for Ireland and all other countries considered costs beyond medication costs. Discount rates were generally not adopted or confidential, except for England and for Ireland in the indications of gating class III and R117H mutations. Overall, detailed information on handling uncertainty was confidential, however, England, Poland and the Netherlands performed deterministic sensitivity analyses and Wales performed a probabilistic sensitivity analysis of patient number and disease management costs. Some countries reported one gross or net budget impact per indication, while others disaggregated their estimates and stated the first and last year budget impact over the chosen horizon.

**TABLE 6:** Overview of budget impact analyses of different European countries for Kalydeco^®^.^a^

Country	Indication	Design						Results^b^
		perspective	time horizon	target population size	costs	discounting	handling uncertainty	
**Belgium** (Rijksinstituut voor ziekte- en invaliditeitsverzekering (RIZIV), 2016a)	Gating class III ≥ 2 years	Payer (RIZIV)	Annual	6 pediatric patients yearly	Medicine-only costs	Not reported	Not reported	Annual budget impact is €1,489,200.
**England **(Whiting et al., 2014)	G551D ≥ 6 years	Payer (NHS England)	total lifetime horizon (3 scenarios) and 1-y horizon;	271 patients, Closed population	costs for genetic testing + Medicine costs, treatment costs directly related to CF, lung transplantation costs	Discount rate of 3.5%on costs	sensitivity analysis scenario analyses	Total additional costs £43,000,000 (year 1)
								The total additional lifetime cost is
								£438,000,000 (conservative scenario) –
								£45QALY (intermediate scenario) –
								£479,000,000 (optimistic scenario)
**Ireland** (National Centre for Pharmacoeconomics (NCPE), 2013a; 2016a; 2017a)	G551D ≥ 6 years	Payer (HSE Ireland)	5 year	113-120 patients (HSE) or 121 (in 2013) to 125 (in 2017) patients (company) Open population	difficult to assess the disaggregated costs for the model as they are not presented in this way	Not reported	Not reported	HSE:
								Gross annual budget impact ranges from €26,532,852 to €28,176,480
								Company:
								Annual net budget impact €28,172,303 (2013) increasing to €28,883,659 in 2017.
	Gating class III ≥ 2 years	Payer (HSE Ireland)	5 year	18 patients Closed population	Not reported	Discount rate of 5%	Not reported	Five-year gross budget impact over €2QALY.
								Five-year net budget impact ranging from €15,300,000 to €22,700,000.
	R117H ≥ 18 years	Payer (HSE Ireland)	5 year	58 patients (year 1) to 65 patients in (year 5) open population	Hospitalization, lung transplantation, medicine acquisition, standard of care (mucolytics, pancreatic enzymes, beta agonists and antibiotics)	Discount rate of 5%	Not reported	Maximum gross budget impact of €13,618,574 (year 1) increase to €15,262,195 (year 5).
								Company:
								The 5-year gross budget impact at €54,055,681 (no net budget impact reported).
									NHS:
									The 5-year gross budget impact may be estimated at €72,554,127
**Poland** Centrum (2014); Agencja Oceny Technologii Medycznych i Taryfikacji (2015)	G551D or gating class III ≥ 6 years	Payer (NFZ)	3 year	9 to 12 patients per year	Direct medical costs: medicine acquisition	No discounting	scenario analysis; sensitivity analysis	Confidential
**Scotland** (Scottish Medicines Consortium (SMC), 2013; SMC, 2016a; SMC, 2016b)	G551D ≥ 6 years	Payer (NHS Scotland)	5 year	53 patients in year 1 to 55 patients in year 5 open population 90% market uptake	Hospitalization, community care, treatment	No discounting	Confidential	The gross impact (same as net impact) on the medicines budget was estimated to be £7,989,000 in year 1 and £8,237,000 in year 5.
	Gating class III ≥ 2 years	Payer (NHS Scotland)	5 year	5 patients in year 1 and year 5 100% market uptake	Medicine acquisition, disease management, hospitalization, lung transplantation	No discounting	Confidential	The net total budget impact was £831,000 in year 1 and in year 5.
								The net total budget impact with savings due to FEV_1_% improvement was £815,000 in year 1 and in year 5.
		R117H ≥ 18 years	Payer (NHS Scotland)	5 year	22 patients (year 1) to 26 patients in (year 5)	medicine acquisition, disease management, lung transplant, adverse events	No discounting	Confidential	Gross budget impact (same as net budget impact) estimated at £4,050,000 in year 1, rising to £4,780,000 in year 5.
	**Sweden **(Tandvårds- och läkemedelsförmånsverket TLV, 2018b)	class III gating mutation	Not reported	Not reported	11 patients	Medicine acquisition	Not reported	Not reported	This would mean a total sale of approximately
SEK 23,000,000 for Kalydeco^®^ in monotherapy in class III gating mutation with applied for AUP.								
The medicine cost (applied for AUP) for Kalydeco^®^ in monotherapy amounts to approximately SEK 2,100,000.								
**The Netherlands** Zorginstituut Nederland (2014)	Gating class III ≥ 2 years	Payer (Dutch National Health Care Institute)	3 year	36 (year 1) – 38 (year 3) or 41 (year 1)- 43 (year 3) (off-label use) open population 100% market penetration market effect: expansion	Medicine acquisition	No discounting	scenario analysis,total patient number increase	Budget impact estimated at €8,900,000- €10,100,000 (off-label) in year one, rising to €9,400,000 - €10,600,000 (off label) in year three.
**Wales **(All Wales Therapeutics and Toxicology Centre, 2013, 2015; All Wales Therapeutics & Toxicology Centre, 2017)	G551D ≥ 6 years	Not reported	5 year	20 (year 1 and year 2) – 21 (year 3) – 22 (year 4) – 23 (year 5) 100% market uptake	Medicine acquisition, administration, monitoring, primary care,secondary & tertiary care, staffing, infrastructure, personal social services	Not reported	Not reported	Confidential
	Gating class III ≥ 2 years	Not reported	5 year	Confidential	Medicine acquisition, secondary & tertiary care	Not reported	Not reported	Confidential
	R117H ≥ 18 years	Not reported	5 year	12 (year 1 and year 2) –	Medicine acquisition, disease management, Liver function test, adverse event	Not reported	Probabilistic sensitivity analysis of patient number and disease management costs	Confidential
				13 (year 3) –				
				13 (year 4) –				
									14 (year 5)
									100% market uptake, mortality rate of 1.5%, 98.9% adherence

a As of June 2021.

b Budget impact results in original currency and year adopted in report (reference in first column).

AUP, average unit price; HSE, Irish Health Service Executive; NFZ, Polish National Health Fund; NHS, National Health Service; RIZIV, Belgian National Institute for Health and Disability Insurance (NIHDI).

##### Orkambi^®^ (Lumacaftor/Ivacaftor)

A BIA was conducted for patients homozygous for the F508del mutation in all selected countries (see Table 7). Calculations were done from the perspective of the payer and the chosen time frame differed, from a 1-year to a 3-year and a 5-year horizon, respectively in Poland, Belgium and the remaining countries. An open population was only considered in Scotland, where patient number dynamically changed with discontinuation and in England, where they accounted for adherence and a yearly incremental market uptake. Other countries considered a closed population. Additionally, Belgium and the Netherlands reported a possible larger population size, in case Orkambi^®^ (lumacaftor/ivacaftor) would expand the current treatment market and be entirely adopted by all ages. Direct medical costs beyond medicine-associated costs, such as hospitalization and adverse events costs, were included in the analyses of England and Ireland only. No information on discount rates was publicly released. Only Poland reported on the use of sensitivity analysis and patient number influencing the potential budget impact. Belgium, the Netherlands and England disaggregated budget impact results and reported yearly amounts. In England, both budget estimates of the company and the national health service were reported, with the latter being slightly higher. One total budget impact estimation over the analyzed time horizon was reported for England, Ireland and Poland.

**TABLE 7:** Overview of budget impact analyses of different European countries for Orkambi^®^.^a^

Country	Indication	Design						Results^b^
		perspective	time horizon	target population size	Costs	discounting	handling uncertainty	
**Belgium **(Zorginstituut Nederland, 2016c)	F508del	Payer (RIZIV)	3 year	336 patients, subgroup analysis (all F508del patients all ages treated): 444 patients	Medicine acquisition	Not reported	Not reported	The annual budget impact is estimated to be €60,400,000 to €79,800,000 in year 1, 2 and 3 depending on population size.
	≥ 12 years			closed population				
	(homozygous)			100% market uptake and compliance				
				Market effect: expansion				
**France **(HAS Service évaluation économique et santé publique, 2016)	Manufacturer was left the choice to perform a BIA but did not include one.							
**England **(NICE, 2015)	F508del ≥ 12 years (homozygous)	Payer (NHS England)	5 year	2,748 patients in year 1 to 2,889 patients in year 5 open population 40% (year 1) to 60% (year 5) market uptake or yearly increment of 5% 90% adherence rate	Direct medical costs: Management, hospitalization, medicine acquisition, liver function test, adverse events	Not reported	Not reported	Company:
								Year 1: £90,273,438
								Year 2: £100,604,425
								Year 3: £110,838,409
								Year 4: £120,855,522
								Year 5: £130,756,207
								The total budget impact over 5 years is £553,328,000.
								NHS:
								Year 1: £92,626,616
								Year 2: £103,226,903
								Year 3: £113,727,659
								Year 4: £124,005,891
								Year 5: £134,164,659
								Total: £567,751,728
**Ireland** (National Centre for Pharmacoeconomics (NCPE), 2016b)	F508del ≥ 12 years (homozygous)	Payer (HSE Ireland)	5 year	505 patients	Direct costs: Medicine acquisition, patient care fee	Not reported	Not reported	Company:
								estimates the 5-year gross budget impact of lumacaftor + ivacaftor at €352,281,736.
								NHS:
								The NCPE estimate of the 5-year budget impact is €391,892,681.
**Poland **(Agencja Oceny Technologii Medycznych i Taryfikacji (AOTMIT), 2018)	F508del ≥ 2 years (homozygous)	Payer	1 year	440 patients closed population	Medicine acquisition	Not reported	sensitivity analysis and scenario analysis most sensitive to total patient number	Annual cost for a public payer amounts to
								PLN 319,950,000 or PLN 727,150,000 per treated patient;
								or PLN 552,640,000 (if 760 patients);
								or PLN 174,520,000 (if 240 patients).
**Scotland** (Scottish Medicines Consortium (SMC), 2019)	Tablets: F508del ≥ 6 years (homozygous) Granules: F508del ≥ 2 years (homozygous)	Payer (NHS Scotland)	5 year	390 patients (year 1) rising to 422 patients (year 5) 100% market uptake 18% discontinuation rate	Confidential	Confidential	Confidential	Confidential
**The Netherlands **(Zorginstituut Nederland, 2016c; 2018; Zorginstituut Nederland, 2019a)	F508del ≥ 12 years (homozygous)	Payers (National Health Care Institute)	3 year	498 patients, subgroup analysis (if all F508del patients, all ages treated): 741 patients closed population 100% market uptake and compliance market effect: expansion	Medicine-only costs	Not reported	Not reported	The annual budget impact is estimated to be €84,400,000 to €125,500,000 in year 1, 2 and 3 depending on population size.

a As of June 2021.

b Budget impact results in original currency and year adopted in report (reference in first column).

BIA, budget impact analysis; HSE, Irish Health Service Executive; NCPE, National Centre for Pharmacoeconomics; NHS, National Health Service; RIZIV, Belgian National Institute for Health and Disability Insurance (NIHDI).

##### Symkevi^®^ (Tezacaftor/Ivacaftor)

BIAs for Symkevi^®^ (tezacaftor/ivacaftor) were performed in the indication of homozygous F508del and/or heterozygous F508del with residual CFTR function mutation (see Table 8). For all countries the payer’s perspective was adopted to estimate the impact while time horizons included 3-year horizons for the Netherlands and Sweden, a 5-year horizon for Scotland and a lifetime horizon in the case of Sweden. An open population was considered in France and Scotland with the latter country also reporting a 100% market uptake while changes in population size incur partly due to discontinuation. Sweden and the Netherlands studied a closed population. Netherlands predicted market expansion and an alternative population size in case of full market uptake of Symkevi^®^ (tezacaftor/ivacaftor) across all ages in the heterozygous indication. With respect to the scope of costs, medicine-only costs were generally considered while direct medical costs beyond medicine-costs, such as follow-up and maintenance costs, were reported in France only. Discount rates were generally not reported and uncertainty in the analyses for France and the Netherlands was addressed by scenarios. The latter, particularly for the Netherlands, was done by alternating treatment compliance rate. Budget impact results were generally confidential, only Sweden and the Netherlands published one total annual estimate over their respective time horizons.

**TABLE 8:** Overview of budget impact analyses of different European countries for Symkevi^®^.^a^

Country	Indication	Design						Results^b^
		perspective	time horizon	target population size	costs	discounting	handling uncertainty	
**France **(HAS Service évaluation économique et santé publique, 2020)	heterozygous F508del mutation and mutation with residual CFTR-function ≥ 12 years	Third-party payer	3 year	402 patients (year 1)	Medicine acquisition, follow-up, maintenance: transplantation, exacerbations, adverse event	No discounting	Scenario analysis	Confidential
				410 patients (year 2)				
				418 patients (year 3)				
				open population				
**Scotland **(Scottish Medicines Consortium, 2019)	F508del ≥ 12 year	Payer (NHS Scotland)	5 year	320 patients (year 1) rising to 347 patients (year 5)	Confidential	Confidential	Not reported	Confidential
	homozygous or heterozygous with one of the following CFTR gene mutation type: P67L/R117C/ L206W/R352Q/A455E/D579G[KA1] /711+3A→G/ S945L/S977F/ R1070W/ D1152H/ 2789+5G→A/ 3272-26A→G/			open population				
	3849+10kbC→T			100% market uptake				
	in combination therapy with ivacaftor 150 mg tablets			13.63% discontinuation rate				
**Sweden **(Tandvårds- och läkemedelsförmånsverket TLV, 2018b)	F508del	Payer (TLV)	Lifetime	Company:confidential	Medicine acquisition	Not reported	Not reported	Confidential
	≥ 12 years							
	Homozygous							
	in combination therapy with ivacaftor 150 mg tablets							
	F508del ≥ 12 years heterozygous + another mutation associated with a residual function in CFTR in combination therapy with ivacaftor 150 mg tablets			20 patients				Total sale of the equivalent of approximately
								SEK 37,000,000 per year.
								The medicine cost for Symkevi^®^ in combination with Kalydeco^®^ amounts to approximately SEK 1,900,000 per patient per year.
**The Netherlands **(Zorginstituut Nederland, 2019b **;** Zorginstituut Nederland, 2020d)	Homozygous F508del	Payer (Dutch National health care institute);	Not reported	250 patients	Not reported	Not reported	Not reported	List price of Symkevi^®^ and Kalydeco^®^ is higher than Orkambi®’s list price.
	≥ 12 years in combination therapy with ivacaftor 150 mg tablets							Budget impact is approximately €3,900,000 in 2019.
	heterozygous F508del mutation and mutation with residual CFTR-function ≥ 12 years	Payer (Dutch national health care institute);	3 year	131 patients,	Medicine acquisition	Not reported	Scenario analysis (compliance)	Total budget impact of
				subgroup analysis (all F508del				€21,326,454 each year –
				patients, all ages treated including off-label use): 153 patients				€24,907,996 each year (subgroup analysis)
				closed population				Sensitivity analysis:
				100% market uptake				Budget impact is
				88% compliance				€24,234,607 (off label use, 22 patients)
								market effect: expansion	and €28,304,541 (100% compliance in year 3)

a As of June 2021.

b Budget impact results in original currency and year adopted in report (reference in first column).

NHS, National Health Service; TLV, Danish Tandvårds- och läkemedelsförmånsverket.

### Managed Entry Agreements

To have CF products reimbursed, the company and some European countries have set up a unique portfolio-deal agreement (Bruce, 2018; Vertex Pharmaceuticals Incorporated, 2018c). The concept was introduced to pay for the company’s CF products considered expensive, not cost-effective and clinically uncertain in many jurisdictions. These portfolio deals aim to facilitate entry of the company’s current products and those in the pipeline for the treatment of CF, while mitigating potential risks for their reimbursement (Rawson, 2018). To that end, a confidential discounted price based on caps, is agreed upon and, in many instances, this contract is coupled with the collection of data concerning clinical uncertainties.

The Republic of Ireland pioneered in 2017, as the first market to establish this portfolio approach for Kalydeco^®^ (ivacaftor) and Orkambi^®^ (lumacaftor/ivacaftor) and the company’s future CF products (Vertex Pharmaceuticals Incorporated, 2017; Vertex Pharmaceuticals Incorporated, 2018c). This agreement, with HSE, formed the blueprint for similar subsequent contracts between the company and Swedish TLV and county councils but also the Danish pharmaceutical and procurement body, Amgros (Nawrat, 2018; Vertex Pharmaceuticals Incorporated, 2018c; Vertex Pharmaceuticals Incorporated, 2018b; Vertex Pharmaceuticals Incorporated, 2018a). A recent study claims that the agreements in Sweden are mostly cost-sharing to address affordability whereas clinical uncertainties usually remain unsolved (Andersson et al., 2020). In Denmark, the price caps in the agreement are linked to the number of patients adopting the treatments (Bruce, 2018). In 2019, the company managed to bring its portfolio approach to England, Northern Ireland and Wales (Vertex Pharmaceuticals Incorporated, 2019c; National Institute for Health and Care Excellence (NICE), 2021). This agreement is performance based and supersedes any previous agreement between the company and NICE (National Institute for Health and Care Excellence (NICE), 2021). Under this deal, the company is required to deliver answers to clinical uncertainties that arose after health technology appraisal. These a priori defined elements and data are collected in the UK CF registry, that is monitored by NICE and funded by the company.

In other markets where reimbursement of the CF products exists, the company has agreed on other proposals.

Switzerland reached an agreement for the eligible population of Orkambi^®^ (lumacaftor/ivacaftor) and Symkevi^®^/Symdeko^®^ (tezacaftor/ivacaftor) along with any future extension by age for Symkevi^®^/Symdeko^®^ (tezacaftor/ivacaftor) (Carvalho, 2020; Plüss, 2020; Vertex Pharmaceuticals Incorporated, 2020). These medicines were added to the Swiss medicine specialties list and are reimbursed by health insurance. This deal could also facilitate future market entry of Kaftrio^®^/Trikafta^®^ (ivacaftor/tezacaftor/elexacaftor) for which an application has been filed with Swissmedic.

In Scotland, a 5-year interim deal for Orkambi^®^ (lumacaftor/ivacaftor) and Symkevi^®^ (tezacaftor/ivacaftor) was realized in 2019, requiring to collect real-world evidence and to resubmit the medicines to the Scottish Medicines Consortium during the contract period (Vertex Pharmaceuticals Incorporated, 2019b; Cystic Fibrosis Trust, 2019). In 2020, a deal for the triple-therapy, Kaftrio^®^ (ivacaftor/tezacaftor/elexacaftor) was reached even before market authorization in Europe (Cystic Fibrosis Trust, 2020).

In 2016, a pay-for-performance agreement was set up between the company and NIHDI due to remaining concerns about high budget impact and effectiveness, in terms of disease progression, survival rates and hospitalization rates (Comissie voor Gezondheid en Gelijke Kansen, 2019; Comissie voor Gezondheid en Gelijke Kansen, 2021). This allowed for a 3-year temporary inclusion of Kalydeco^®^ (ivacaftor) on the Belgian reimbursement list. In return, the company was required to collect data and resolve established clinical uncertainties (Fair Healthdata, 2015; Sectoraal Comité, 2017). To account for the budgetary risks, a yearly amount based on profits and number of treated patients was refunded to NIHDI (Rijksinstituut voor ziekte-en invaliditeitsverzekering (RIZIV, 2016a; RIZIV, 2016b). Since the end of the agreement, it has been amended, renewed and is still ongoing (Rijksinstituut voor ziekte-en invaliditeitsverzekering (RIZIV, 2019). An agreement for the reimbursement of cystic fibrosis medicines, Orkambi^®^ (lumacaftor/ivacaftor) and Symkevi^®^ (tezacaftor/ivacaftor), was reached in March of 2021 (Mucovereniging, 2021; Vlaamse Radio-en Televisieomroeporganisatie (VRT), 2021). That same month, the company applied for reimbursement of their most recent innovative therapy, Kaftrio^®^ (ivacaftor/tezacaftor/elexacaftor).

In the Netherlands, although not cost-effective, Orkambi^®^ (lumacaftor/ivacaftor) was added to their reimbursement list (van Rijn, 2016). Currently, a confidential price-agreement with conditions is set up between the company and the government for all three modulators (Zorginstituut Nederland, 2019a; Zorginstituut Nederland, 2019b; Zorginstituut Nederland, 2021a). Likewise, for Orkambi^®^ (lumacaftor/ivacaftor), a straight reimbursement deal was achieved in Austria but also Kalydeco^®^ (ivacaftor) and Symkevi^®^ (tezacaftor/ivacaftor) are found on their specialty list (Pinto, 2018; Rawson, 2018; Vertex Pharmaceuticals Incorporated, 2018a; Österreichische Sozialversicherung, 2020).

Both Kalydeco^®^ (ivacaftor) and Orkambi^®^ (lumacaftor/ivacaftor) are reimbursed in France (Vertex Pharmaceuticals Incorporated, 2019). Kalydeco^®^ (ivacaftor) was given a positive decision after reimbursement application (Haute Autorité de Santé HAS, 2014). For 4 years, Orkambi^®^ (lumacaftor/ivacaftor) was available to a set of patients through a temporary use authorization (ATU) until a price agreement was achieved (Association Gregory Le Marchal - Vaincre la Mucoviscidose, 2019). Recently, Symkevi^®^ (tezacaftor/ivacaftor) price negotiations were finalized and the medicine was added to the reimbursement list (Journal Officiel de le République Française, 2021; La Voix Du Nord, 2021; Vertex Pharmaceuticals Incorporated, 2021).

Since the market authorization of the cystic fibrosis medicines by the European Commission, the modulators are available in Germany (GKV-Spitzenverband, 2012; GKV-Spitzenverband, 2015; Vertex Pharmaceuticals Incorporated, 2016; GKV-Spitzenverband, 2018a). However, a reimbursement agreement between the German National Association of Statutory Health Insurance Funds (GKV) and the company was founded on the obligation of the pharmaceutical company to automatically report the CF modulators’ price and product information through electronic data transmission, in accordance with legal Section 131 (4) SGB V (GKV-Spitzenverband, 2018b).

In Spain, managed entry of Orkambi^®^ (lumacaftor/ivacaftor) and Symkevi^®^ (tezacaftor/ivacaftor) in combination with Kalydeco^®^ (ivacaftor) was obtained by establishing a mixed model of financing (Rivera, 2019). Spanish health authorities agreed on the company’s proposal for a cap on spending combined with pay-for-performance reflecting clinical uncertainty (Vertex Pharmaceuticals Incorporated, 2019a; Grubert, 2019).

### International Collaborations

Several countries were hesitant to adopt Orkambi^®^ (lumacaftor/ivacaftor) due to its high price and clinical uncertainties. To mitigate these uncertainties, Belgium and the Netherlands performed a joint price negotiation as part of the Beneluxa initiative in 2015 (O'Donnell, 2015; Paun, 2018; Rawson, 2018; Beneluxa Initiative on Pharmaceutical Policy, 2021). The negotiation was a pilot study of a larger international collaboration, additionally involving Luxembourg, Austria and Ireland, which was set up to jointly assess highly priced and innovative medicines often intended for a small population (De Block, 2015). In the case of Orkambi^®^ (lumacaftor/ivacaftor), Belgian and Dutch negotiations resulted in a negative decision to reimburse the medicine as no agreement could be established (Allen, 2017). The Ministers of Health deemed the medicine to be overpriced and not cost-effective (van Rijn, 2016; De Block, 2017). A price reduction of 82% was requested to make Orkambi^®^ (lumacaftor/ivacaftor) cost-effective. Ultimately, Netherlands managed to strike a deal with the company alone. Another 4 years was needed for Orkambi^®^ (lumacaftor/ivacaftor) to be reimbursed and available in Belgium (Zorginstituut Nederland, 2017; Mucovereniging, 2021).

## Discussion

Kalydeco^®^ (ivacaftor), Orkambi^®^ (lumacaftor/ivacaftor) and Symkevi^®^ (tezacaftor/ivacaftor) were the first treatments authorized in the European Union to target the underlying mechanism of the dysfunctional *CFTR* protein in cystic fibrosis (Lopes-Pacheco, 2016; Rafeeq and Murad, 2017; Lopes-Pacheco, 2019). With these treatments and the latest Kaftrio^®^ (ivacaftor/tezacaftor/elexacaftor), about 90% of PWCF are able to be treated, however access to these CFTR modulators in EU Member States is challenged because of the associated high costs and constricted healthcare budgets (Schneider et al., 2017; Rawson, 2018).

### Prices and Efficacy

Although, prices are closely relatable in some countries, our findings show that Kalydeco^®^ (ivacaftor) is generally the most expensive CFTR modulator, followed by Symkevi^®^ (tezacaftor/ivacaftor) and, lastly, by Orkambi^®^ (lumacaftor/ivacaftor). This price relation may be reflective of the effectiveness of the modulators, as Kalydeco^®^ (ivacaftor) has proven to be of highest clinical added value. At the launch of Kalydeco^®^ (ivacaftor) unmet medical need for PWCF was high and no alternative was available. The modulator showed significant lung improvement in gating mutations but was indicated for a small population. (Lopes-Pacheco, 2019; European Medicines Agency, 2021b). More recently, an observational study confirmed the ability of treatment with Kalydeco^®^ (ivacaftor) to be disease modifying (Bessonova et al., 2018). With the introduction of Orkambi^®^ (lumacaftor/ivacaftor), the most common mutation in PWCF was able to be treated and clinical studies showed moderate lung function amelioration, however the tolerability of this treatment in patients with low baseline lung function was poor and interactions with other medication had been reported. With Symkevi^®^ (tezacaftor/ivacaftor) a more extensive population is able to be treated with comparable but fewer side effects than Orkambi^®^ (lumacaftor/ivacaftor) (Lopes-Pacheco, 2019). Health authorities might have had greater negotiation power and might have been stricter on price depending on clinical added value and unmet medical need with the second and third generation of medicines, namely Orkambi^®^ (lumacaftor/ivacaftor) and Symkevi^®^ (tezacaftor/ivacaftor).

Furthermore, our results highlight apparent intra-variability when it comes to pricing of the same medicine in different European countries. As price-setting and HTA in Europe is determined nationally by the member states, price differences are inevitable (Young et al., 2017). It should also be noted that reported list prices may differ from the actual paid price subject to a discount determined in a confidential contract with the company. This discount differs among countries and is dependent on factors such as the country’s negotiation power and use of external reference pricing (Rémuzat et al., 2015). Additionally, prices for individuals may vary from these averages, as dosage differs according to weight and age group. Underlying price differences may also be influenced by the included pharmacist fees, wholesale quotas and the national VAT on prescription-only medicines.

### Economic Evaluations

Overall, Kalydeco^®^ (ivacaftor), Orkambi^®^ (lumacaftor/ivacaftor) and Symkevi^®^ (tezacaftor/ivacaftor) were considered not cost-effective in the studied countries. HTA bodies unanimously reported clinical uncertainties on long-term lung function and requested a price reduction of the modulators for the ICER to fall below the adopted threshold or comply with their cost-effectiveness requirements. Some countries (Netherlands, England, Sweden, France) questioned the accuracy of the ICER values determined by the company and requested additional data to support their outcome or delivered a recalculated ICER value showing the company’s initial ICERs to be a considerable underestimation. Apparent discrepancies between ICER values internationally could be explained by differences in methodological guidelines for economic evaluations (Hay et al., 2010). The chosen simulation model, patient-level microsimulation or Markov, but also differences in perspective, payer or society, could affect the outcome (Schuller et al., 2015). Furthermore, differing ICERs could be influenced by the source for retrieval, from clinical trial or country-specific data, of input values such as QALYs or costs, particularities in healthcare system and the applied discount rate.

Cost-effectiveness might also be influenced by the approach adopted for the assessment of these CFTR modulators. In Poland and Ireland, Kalydeco^®^ (ivacaftor) and Symkevi^®^ (tezacaftor/ivacaftor) were assessed in their general HTA process for medicines and under the same criteria and threshold as non-orphan medicines (Caban et al., 2016; National Centre for Pharmacoeconomics (NCPE), 2017b; Vaithyanathan et al., 2018; Malinowski et al., 2019). It was shown that with these conditions, orphan medicines are most likely not to be cost-effective due to typical characteristics of a small population and limited clinical data availability. Some states, such as England, Scotland and Wales, established a HTA process specific to orphan medicines and others, like Sweden and the Netherlands, rely on a process dependent on disease severity, which allowed Kalydeco^®^ (ivacaftor) and Symkevi^®^ (tezacaftor/ivacaftor) to be measured against a higher or more flexible ICER threshold (Denis et al., 2009; NHS England, 2015; National Institute for Health and Care Excellence (NICE), 2017; Kelly et al., 2018; Tandvårds-och läkemedelsförmånsverket TLV, 2019). Other countries like Belgium, France and Germany do not rely on ICER values to determine the value of (orphan) medicines (Denis et al., 2009). In Belgium, the company was exempt of delivering a cost-effectiveness analysis and only a BIA for both orphan medicines was requested. In France, the CF modulators were evaluated according to their clinical added value or service medical rendu (SMR) and similarly in Germany, the assessment was based on additional medical benefit (Denis et al., 2009; IHS Global, 2013; Gerber-Grote et al., 2014; Haute Autorité de Santé HAS, 2014; Haute Autorité de Sant é , 2019; Haute Autorité de Sant é , 2020c).

### Budget Impact Analyses

Budget impacts varied amongst countries and were dependent on the country’s CF patient number, medication prices, included or excluded treatment-related costs and discounting. Comparison of budget impact between countries and interventions was complicated as results were reported over varying time horizons and numerical outcomes were not depicted in a consistent form. Some countries such as England and Germany, considered a budget impact threshold (Ollendorf et al., 2017; Institut für Qualität und Wirtschaftlichkeit im Gesundheitswesen (IQWIG), 2020; IQWIG, 2021). In England this meant that commercial discussions were mandatory for reimbursement of Kalydeco^®^ (ivacaftor) and Orkambi^®^ (lumacaftor/ivacaftor). Germany did not release information on their budget impact calculations, however benefit reassessment by their health authority, G-BA, meant that both orphan medicines Kalydeco^®^ (ivacaftor) and Symkevi^®^ (tezacaftor/ivacaftor) breached their €50 million budget impact benchmark (Institut für Qualität und Wirtschaftlichkeit im Gesundheitswesen (IQWIG), 2020; IQWIG, 2021). Overall, countries unanimously considered budget impact to be high. The accuracy of the budget estimates is not guaranteed as analyses did not always methodologically adhere to BIA guidelines, information was missing and some parameters were unspecified (Sullivan et al., 2014). Lack of transparency due to confidentiality also prevents insight into the actual budget impact. Assuring the methodological quality of future BIA could allow a more in-depth analysis and better informing of decision-makers on affordability (Abdallah et al., 2021).

### Managed Entry Agreements

The reimbursement of the CF products was possible through MEAs between specific countries and the company. HTA reports emphasized not only the need to reduce prices substantially to increase affordability but also to address uncertainty around long-term clinical efficacy. To resolve uncertainties, some countries conditioned the reimbursement by requiring the company to monitor medicine administration and collect data on agreed efficacy measures in a register. Conventionally, these agreements are temporary, revised periodically and put in place for one product. In this case, the company pioneered with their portfolio-deal agreement for all their current and future CF products. The impact of this type of agreement on affordability and evidence collection is still uncertain, but arguably, agreeing to reimburse all future CF products without a rigorous HTA might have critical implications in the future.

### Cross-Border Collaboration

Although the Netherlands and Belgium joined forces in price negotiations for reimbursement as a pioneer project under the Beneluxa initiative, an agreement could not be reached (Allen, 2017). To accommodate a seamless market access process for high cost and innovative medicines in the future, efforts towards information sharing and joint assessment such as done by Beneluxa and the International Horizon Scanning Initiative, should be maintained and further developed (Natsis, 2019; Beneluxa Initiative on Pharmaceutical Policy, 2021). Expansion in terms of number of countries participating in such initiatives should be further encouraged, as coalitions for negotiations with pharmaceutical companies have proven to be successful (Government of the Netherlands, 2018; Sheet, 2019). Moreover, to circumvent intricacies relating to various HTA processes amongst countries, performing assessments aggregately in an independent, joint network such as EUnetHTA could promote a more streamlined process. In turn, this could equip countries with more reliable, transparent and qualitative information to accurately perform their national HTA and increase their bargaining power with companies (O’Mahony, 2019; European Network For Health Technology Assessment, 2021).

Our study shows that despite failed cost-effectiveness, high budget impact and negative recommendations, Kalydeco^®^ (ivacaftor), Orkambi^®^ (lumacaftor/ivacaftor) and Symkevi^®^ (tezacaftor/ivacaftor) are reimbursed in the majority of analyzed countries. MEAs and portfolio deals allowed for the adoption of these CF medicines but also other decision criteria such as equity and equal access, disease severity, innovation, patients’ and clinicians’ views, patient advocacy, media attention but also prevalence seem to have played a role in final reimbursement decisions (Denis et al., 2009; Drakeford, 2013; National Institute for Health and Care Excellence (NICE), 2017; Ollendorf et al., 2017; Kelly et al., 2018; Tandvårds-och läkemedelsförmånsverket TLV, 2019; Andersson et al., 2020; Smith and Barry, 2020; Scottish Medicines Consortium, 2021b).

### Strengths and Limitations

Our study sheds light on the market access of CFTR modulators in European countries based on a comprehensive analysis of pricing information, economic evaluations, BIAs, MEAs and reimbursement decisions. However, our findings were limited by public availability of data and confidentiality of reports. Depicted prices are facial prices and do not reflect the actual medicine price with discount. Critical information on cost-effectiveness and budget impact was often blacked-out or assessment reports were incomplete. Thus, the selection of countries in this study was based on availability of HTA documents. Little insight of MEAs was possible, therefore, details on considered clinical uncertainties and their influence on the final agreed discount is unknown.

## Conclusion

This study shows that the CFTR modulators Kalydeco^®^ (ivacaftor), Orkambi^®^ (lumacaftor/ivacaftor) and Symkevi^®^ (tezacaftor/ivacaftor) are generally considered to be expensive, not cost-effective and with a high budget impact in selected European countries. Reimbursement of these medicines was dependent on the ability of respective countries to form an agreement with the company. Even though most analyzed countries offered full reimbursement of treatments, some only selectively reimbursed certain treatments (Sweden) or none at all (Poland). Our findings point to unequal access, differential pricing and delayed availability of cystic fibrosis modulators in Europe.
